# Supplementary material for: Performance Evaluation of Microscanner Plus, an Automated Image-Based Cell Counter, for Counting CD4+ T Lymphocytes in HIV Patients
Source: Diagnostics (Basel). 2023 Dec 28;14(1):73. doi: 10.3390/diagnostics14010073 (PMC10871079; doi:10.3390/diagnostics14010073)
Supplement: Supplementary file 1 [file diagnostics-14-00073-s001.zip › diagnostics-2767948-supplementary.pdf]

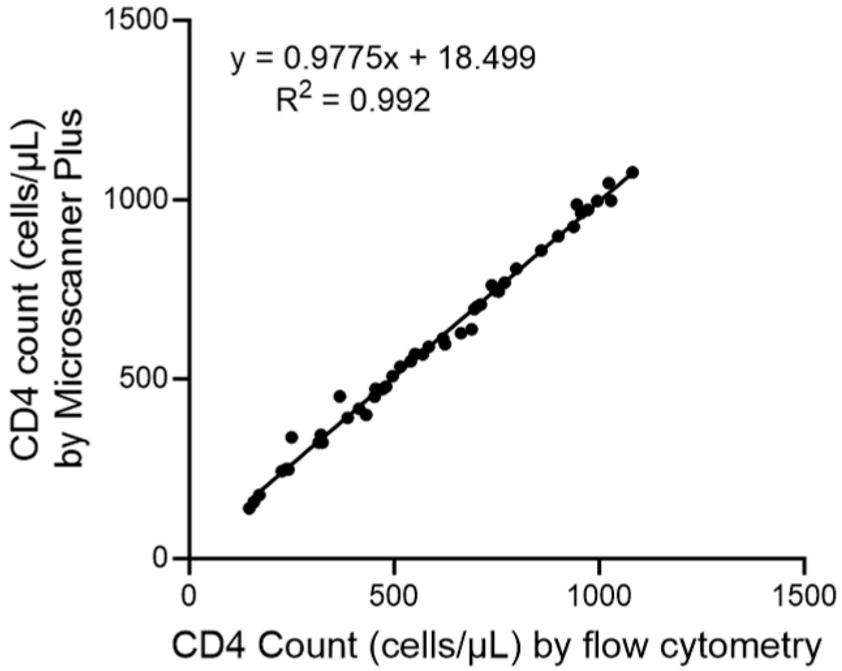

Figure S1. Correlation of CD4 counts measured by Microscanner plus (MSP) and Flow cytometry (FCM) for 46 clinical samples with a lymphocyte/WBC ratio of less than 20%.

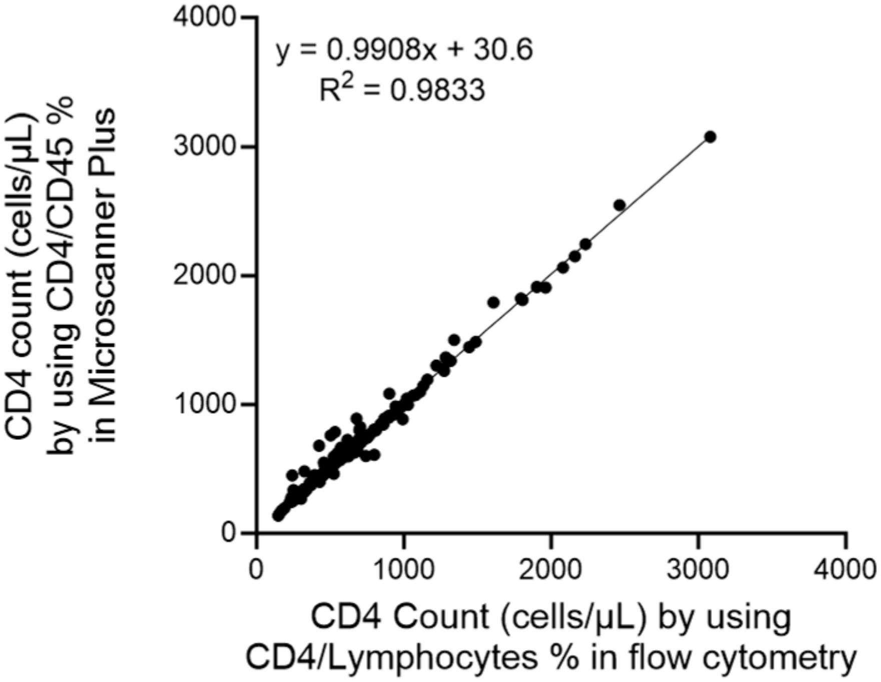

Figure S2. Correlation of CD4 counts measured by Microscanner plus (MSP) with CD4/CD45% and Flow cytometry (FCM) with CD4/Lymphocytes % for 134 clinical samples.
